# Extinction studies across the disciplines

**DOI:** 10.1017/ext.2024.23

**Published:** 2024-12-05

**Authors:** Graham Huggan, Alison M. Dunn, Diane Nelson, Katy Wright, Dolly Jørgensen, Kate Rigby

**Affiliations:** 1 University of Leeds, Leeds, UK; 2 University of Stavanger, Stavanger, Norway; 3 University of Cologne, Cologne, Germany

Extinction is nothing if not a multivariate and “polysemous” concept; despite the finality it appears to confer on those affected by it, it has different temporalities attached to it, and has meant broadly different things to different people at different times (O’Key 2023: 168; see also Jørgensen, 2022, Van Dooren, 2014). As recently as a couple of centuries ago, the idea that species could disappear was widely considered to be improbable (Wignall, 2019: 1); today, the reality that they *do* disappear, and that those disappearances have potentially devastating consequences, has percolated into public life (O’Key, 2023: 172–173).

Although paleobiologists do not necessarily agree on this, the Earth today is experiencing what is popularly known as the Sixth Mass Extinction (Barnowsky et al., 2011; for a different view, see also Bambach et al., 2004). Over a million of the world’s animal and plant species are currently considered to be under serious threat, while our long-term future on the planet is increasingly uncertain. Meanwhile, the recent COVID-19 pandemic has reminded us that humans, animals and even pathogens are co-dependent on each other’s survival; that our deaths are shared with others’ deaths, just as our lives are entangled with others’ lives (Van Dooren, 2014: 137).

Unsurprisingly given this context, a new field, Extinction Studies, has emerged over the past decade or so – mainly in Australia and the US, but now increasingly in the UK – that directly engages with the questions raised by these circumstances. Extinction Studies, as it is currently conceived, tends to be either biologically or culturally oriented; in this special issue, and the CUP Extinction series that hosts it, we make a strong case that it needs to be both. Extinction needs to be understood, that is, in *cultural* as well as *biological* terms, not least through the various stories that are told about it; and also through the various languages that are associated with it, which are as much tied up with emotions as with ideas, with cultural values as with scientific facts (Heise, 2015). Extinction, as the cultural theorist Ashley Dawson suggests, may well be an “under-acknowledged form – and cause – of the contemporary environmental crisis”, but it is equally a crisis of the *imagination*: a product of our seemingly impoverished capacity to conceive of a more socially equitable, biologically diverse world (2016: 9; 101).

The main question the contributors to this issue have been instructed to ask is what extinction means – biologically, culturally, socially – in contemporary contexts of global crisis: the decline of species, the death of languages, the deterioration of already vulnerable human societies and natural ecosystems. Further questions arise from this. What is the future of life in a time of mass extinctions? What is our place in, and what are our obligations to, a more-than-human world: one that we humans share with a multitude of other species whose lives are bound up with our own? What are the different meanings of extinction in different social and ecological contexts, and what stories and narratives, as well as everyday material practices, are needed to cope with the unprecedented scale of loss? Can extinction be forestalled, or even reversed, and what are the consequences of such measures? What legacies does extinction leave us with, and what does it tell us about the all too easily taken-for-granted temporal processes – generational change, the death-life continuum – that it “catastrophically interrupts”? (Van Dooren et al., 2017: np).

Addressing extinction requires collaborative work: across species, across nations, across societies and cultures, but also across *disciplines.* Extinction Studies is a field that by definition crosses conventional disciplinary boundaries, creatively combining the work of arts- and sciences-based researchers. It is also one that by definition attracts the young, on whose own futures the future health and biodiversity of the planet depend. This special issue is dedicated, accordingly, to the work of early-stage researchers based at universities in the UK and Europe. Although their submissions were made anonymously and blind-reviewed, around half of the contributors to the issue are affiliated with the UK’s pioneering doctoral scholarships program at the University of Leeds in Extinction Studies, researchers attached to which are required to work in and across at least two different disciplines^1^; while the other contributors, who are currently based at various European universities, are pursuing similar kinds of interdisciplinary work. In some cases, especially those of the Leeds-based contributors, essays have been co-authored with more experienced university colleagues, including their own supervisors, but the collaborative work that has been produced is very much led by younger scholars who are laying the foundations, not just for their own professional futures but for the intellectual and institutional future of the field.

It would be mistaken, for all that, to see Extinction Studies as a field that is directed inexorably toward the future. On the contrary, as the essays collected here amply demonstrate, it requires a greater understanding of the past or, better, of multiple pasts: of the histories that underlie human encounters with (other) animals; of the histories that underscore particular nations’ engagements with industrial modernity, or particular peoples’ understandings of the natural world; of the still longer histories that inform, and indeed precede, human beings’ presence on Earth. In this and other ways, Extinction Studies is not just a study of loss, or of the many different ways that we might seek in future to counteract it; it is a study of temporal processes, not least the grand narrative of evolution itself. Extinction Studies, in sum, is an inclusive field even as its subjects are cruelly excluded from the future; and it is a field that demands sharpened historical awareness even as its subjects are prematurely consigned to the past. Finally, it is a field that actively requires the bringing together of knowledge from different places, different times and different research areas – a field that is as alive as it gets even as its subjects have reached the narrative endpoint of their lives.

The essays gathered in this special issue contain several overlapping themes and do not require to be read in any particular order; however, for ease of reference, we have arranged them into three more or less self-evident groups. These groups correspond to three broad strands of current debate around the study of extinction. The first strand concerns what might loosely be called the *epistemology* of extinction. As the lead-off piece by Serena Turton-Hughes and her Leeds-based colleagues indicates, the causes and drivers of extinction are probably better known today than they have ever been; but despite this many extinctions remain wholly unknown to us, and many species have already disappeared (or are in the process of disappearing) without our knowledge, and without our having been able to take the opportunity to study them, classify them and protect them should the need arise. And as the piece that follows it, by the Oulu-based postdoctoral researcher Eline Tabak, suggests, the classification of species can itself be seen as an epistemic problem in so far as the scientific study of species, along with the taxonomies that arise from it, is far from ideologically unmotivated or value-free.

The other essay that makes up this first strand, a collaborative piece by literary scholar Alfie Howard and linguist Diane Nelson, discusses the further political implications of these epistemological uncertainties. The literature, as Howard and Nelson show, is as good a vehicle as any for the exploration of some of the fundamental sources of uncertainty in Extinction Studies. These include whether endangered entities will eventually go extinct, whether we can predict when this might happen, and whether such predictions can bring about the sorts of actions by which their extinction can be prevented – if this prevention is felt to be necessary in the first place; and as if biological and cultural diversity were automatically worth upholding, which is itself a deeply political debate. The extinction of languages, one of the subjects of Howard and Nelson’s essay, is a good example of what might broadly be called the political epistemology of extinction: the different ways in which knowledge of extinction threats can be instrumentalized for political purposes that are by no means egalitarian and progressive. This reminds us that extinction is “both a material reality and a cultural discourse that shapes popular perceptions of the world” (Dawson, 2016: 15); and that it is often complicit with an “inegalitarian social order” which justifies the losses it produces as much as the surpluses it creates (Dawson, 2016: 15; see also Mitchell, 2024).

Dawson’s explicitly anticapitalist approach is matched, to some extent at least, in the four essays that make up the second strand of the issue, all of which focus on the *discourse of species*, Cary Wolfe’s resonant term for that nexus of socially constructed knowledge formations through which species are popularly as well as scientifically understood (Wolfe, 2003). The first two essays attached to this strand, by the Oslo-based anthropologists Sonja Åman and Marius Palz, discuss governance issues surrounding the conservation of critically endangered marine mammals (North American right whales and Japanese dugongs, respectively); the third, by Sicily Fiennes and her colleagues at the University of Leeds, dissects the illegal wildlife trade that poses an existential threat to rare species of Asian songbirds; while the fourth, by the Bayreuth-based doctoral researcher Eleanor Schaumann, traces transverse historical connections between settler colonialism, genocide and the preservation of Namibian Karakul sheep. In each case, the essays take a multispecies approach to the social and (bio)political conflicts involved in the perception and management of these severely threatened rather than technically extinct creatures. They also explore the contradictions embedded within ongoing attempts to protect them, drawing on now well-documented debates surrounding the imbrications of conservation and colonialism, both of these with industrial capitalism, and all of these with discourses of racism, sexism and speciesism that legitimate the dominance of certain species over other species, and assert the “hyperseparation” of human beings from the rest of the natural world (Plumwood, 1993; see also Duffy, 2010, Mitchell, 2024).

The third and last strand of the issue is arguably its most creative. Featuring innovatively cross-disciplinary essays by Kate Simpson (creative writing/philosophy/ paleontology) and Jonathan Roberts (history/biology/medical science), and rounded off by a collaborative photo essay by researchers associated with the University of Leeds’s aforementioned Extinction Studies doctoral training program, the focus of this particular strand is on the *spatiotemporal* dimensions of extinction: on the place-based pathologies that inform it; on the pre- and geohistorical narratives that are connected with it; and on its situated engagements with deep time. These last three essays also help cement the case for a suitably imaginative, consciously interdisciplinary approach to extinction and the issues, both practical and theoretical, that surround it. Much (perhaps too much) has been said and written about the virtues of interdisciplinarity in addressing the challenges of our times, as well as in achieving a greater understanding of the historical conditions that produced them. The possibility of our own extinction, along with that of countless other species, is perhaps the greatest of these challenges. However, maybe the most pertinent thing that can be said about the study of extinction today is that it requires a collective understanding of human accountability: one we all need to own up to even if the material exigencies attached to our current predicament are unevenly shared.

As Thom van Dooren argues, extinction involves more than the documented disappearances of multiple species and the potential disappearances of many others; it also challenges us to imagine the losses of what these species might have become (2014: 38–39). As such, extinction implies a radical foreshortening of the future: of our own human future, to be sure, but also the many possible futures of the creatures with whom we share the planet and on whose lives our own lives depend. Its study is thus an ethical imperative as well as a stimulus for action. Extinction, Dominic O’Key evocatively suggests, is a “haunting which follows our actions”; to understand it better in the future may eventually be to find a way to “disabuse [ourselves] of [the] desire for mastery” that has led to many, though by no means all, extinctions in the past (2023: 29). And if a better understanding of extinction requires empathy and humility, it also requires the connective power of the imagination. For if each extinction event must be seen and recognized in its own terms, extinction is not a singular phenomenon (Van Dooren 2014: 147). It and its effects are legion, as the multifaceted essays in this special issue powerfully attest.

## Footnotes

1 We wish to thank the Leverhulme Trust for supporting this program. Some wording from this Editorial can be found in the Trust’s 2020 Annual Review, and we give further thanks for their permission to reprint material here.
